# Lower Extremity Musculoskeletal Complications of Spastic Cerebral Palsy

**DOI:** 10.26502/josm.511500196

**Published:** 2025-04-21

**Authors:** Alexander Abdou, Eli Spector, Sonam Sidhu, Devendra K. Agrawal

**Affiliations:** Department of Translational Research, College of Osteopathic Medicine of the Pacific, Western University of Health Sciences, Pomona, California 91766, USA

**Keywords:** Bony deformities, Cerebral palsy, Lower extremity, Motor dysfunction, Muscle spasticity, Muscle weakness, Musculoskeletal complications, Scoliosis, Shortened muscle-tendon units, Spastic Cerebral Palsy

## Abstract

Spastic cerebral palsy (CP) frequently results in lower extremity musculoskeletal complications that cause disabling mobility loss and reduced quality of life. This review consolidates current understanding of these common complications, encompassing bony deformities (e.g., scoliosis, femoral anteversion, patella alta, ankle equinus), joint changes due to spasticity and contractures (e.g., hip subluxation, knee flexion contractures), spinal degeneration (e.g., cervical stenosis, lumbosacral spondylolisthesis), and neurologic dysfunction manifesting as pain and weakness. These pathologies are generally progressive, driven by impaired motor control, muscle spasticity, and shortened muscle-tendon units. By providing a comprehensive framework, this paper highlights the critical points facilitating a healthcare provider to recognize and understand these complications, ultimately improving patient care and outcomes.

## 1. Introduction

Cerebral palsy (CP) is the most common cause of disability in childhood and contributes to one of the largest groups of people living with disability worldwide [1,2]. CP is a diverse group of permanent and non-progressive disorders defined by disturbance in movement and postural function in the early stages of brain development [3,4]. These disturbances, occurring in the fetal or infant brain, produce variable and durable motor abnormalities and may also present with a wide range of sensory, cognitive, communicative, and behavioral deficits.

## 2. Epidemiology and Risk Factors for Cerebral Palsy

Cerebral palsy is estimated to be diagnosed in 2 to 3 out of every 1000 live births in the United States [5]. Risk factors for CP have frequently been categorized by the timing of the initial neurologic insult into antenatal, perinatal, and postnatal contributors. For antenatal causes of CP, congenital brain malformations, such as schizencephaly and agenesis corpus callosum, carried the highest risk [2,3,6]. However, notably, the most common pathologic imaging finding of those with spastic CP was intracerebral hemorrhage and periventricular leukomalacia rather than a brain malformation [6,7]. To this point, perinatal risk was most significant with incidence of neonatal encephalopathy and subsequent hypoxic-ischemic encephalopathy [3,7,2]. Infections of the central nervous system, followed closely by cerebrovascular accidents, increase the risk most of CP postnatally [2,3].

**Figure 1:**
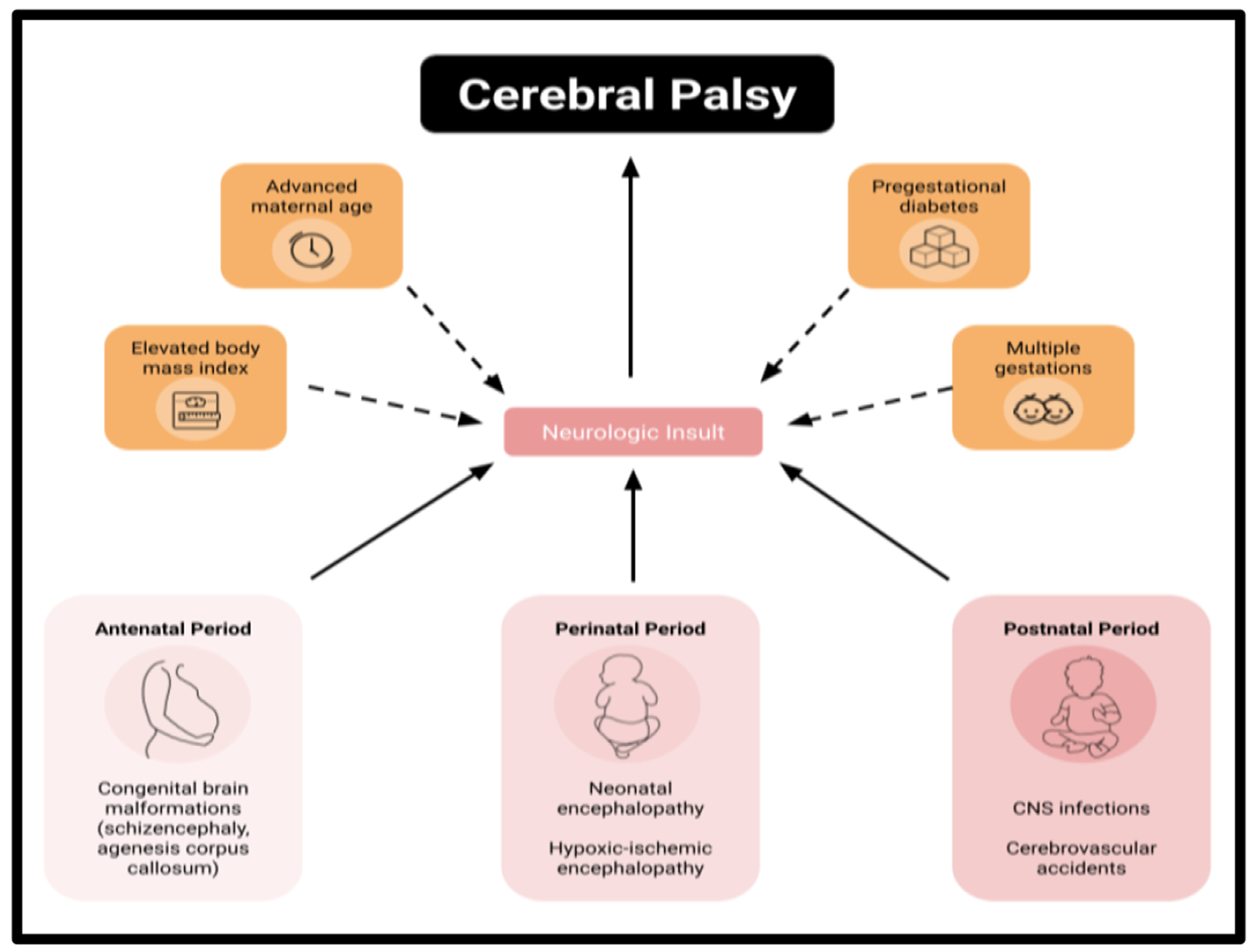
Cerebral palsy results from multifactorial neurologic insult.

Before conception, the maternal environment can predispose a future fetus to developing CP depending on factors such as maternal age, body mass index (BMI), pregestational diabetes, and multiple gestations. Advanced maternal age, defined as pregnant mothers older than age 35, correlated with a higher incidence of CP than other age groups [8]. Regarding overall maternal health, each unit increase in maternal BMI was found to increase the risk of CP by 3% with a clear positive correlation [9]. In addition, when compared to gestational diabetes, maternal pregestational diabetes had an increased risk for CP that was not explained by increased fetal size and subsequent trauma during delivery [10]. Genetic and familial history also play a role with evidence that multiple gestations, with twins and even more so with triplets, increase the incidence of CP [11–14]. These factors predispose the maternal environment toward a neurologic insult that could cause CP in a developing fetus or infant (Figure 1).

Therefore, interventions to manage modifiable risk factors for CP include vitamin K administration to prevent intracranial hemorrhage, prevention of maternal infection via adequate vaccination and screening, early management of hyperbilirubinemia, and delaying delivery until after 28 weeks’ gestation when possible [3,5,14]. The other risk factors, such as elevated BMI, maternal diabetes mellitus, prolonged rupture of membranes, pre-eclampsia, and low fetal birth weight, can be further modified with interventions such as smoking cessation education, substance use programs, and nutrition management [3,5].

Therefore, interventions to manage modifiable risk factors for CP include vitamin K administration to prevent intracranial hemorrhage, prevention of maternal infection via adequate vaccination and screening, early management of hyperbilirubinemia, and delaying delivery until after 28 weeks’ gestation when possible [3,5,14]. The other risk factors, such as elevated BMI, maternal diabetes mellitus, prolonged rupture of membranes, pre-eclampsia, and low fetal birth weight, can be further modified with interventions such as smoking cessation education, substance use programs, and nutrition management [3,5].

## 3. Pathogenesis of Cerebral Palsy

**Figure 2:**
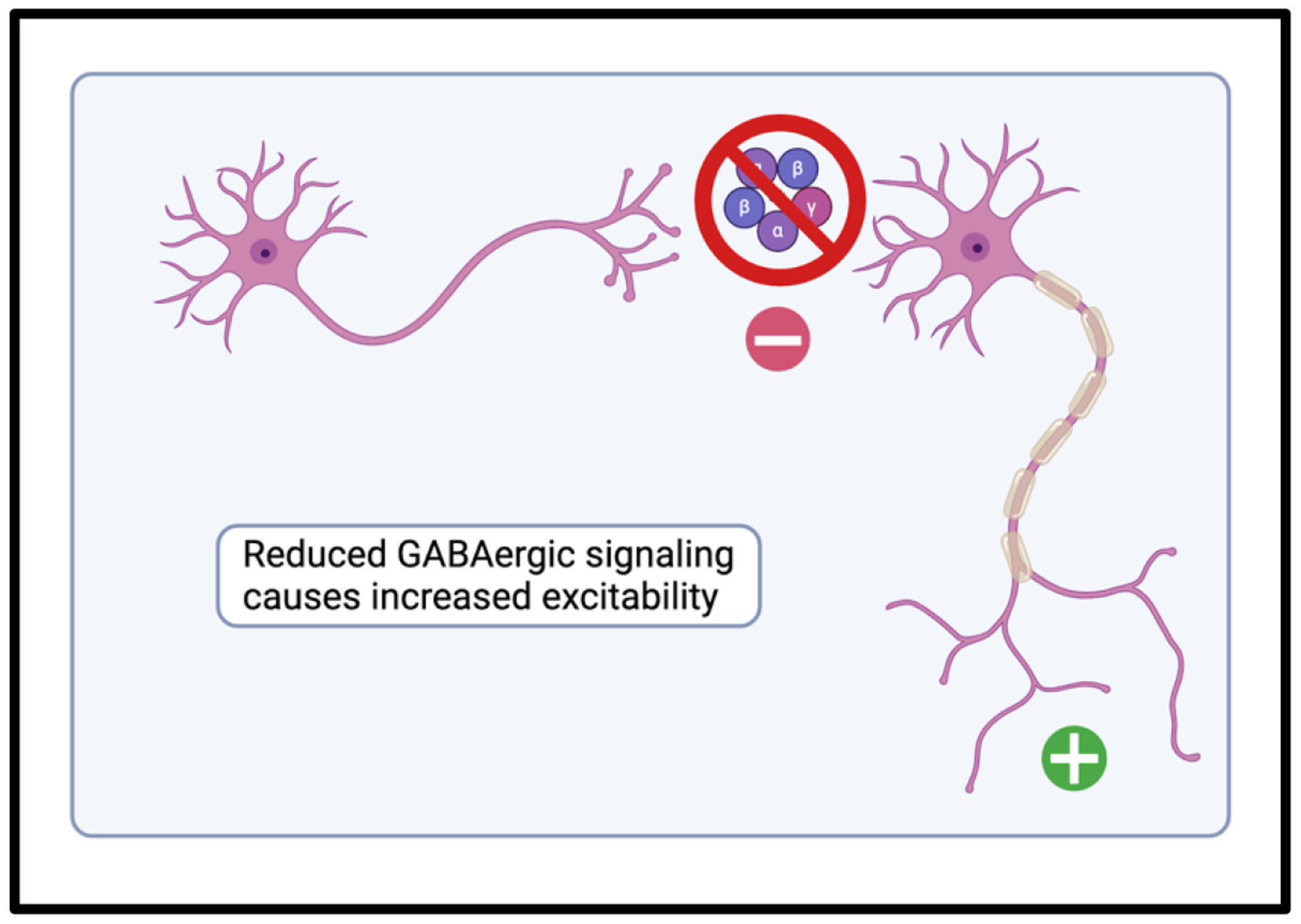
Normal GABAergic signaling in the spinal cord and brainstem acts as an inhibitory stimulus to action potential generation. In spastic cerebral palsy, this signal is reduced, contributing to spasticity.

The pathogenesis of CP is likely multifactorial, related to a variable combination of fetal brain malformation, fetal hypoxemia, and loss of normal inhibitory GABAergic signaling in the spinal cord [15] (Figure 2). CP is clinically categorized into five subtypes, the most common of which is the spastic subtype [16]. Spastic CP significantly impairs independent completion of activities of daily living (ADLs), contributes to chronic pain, and increases healthcare spending [17,18].

The musculoskeletal complications of CP can be categorized into four major neuromuscular impairments, including impaired motor control, muscle spasticity to stretch, shortened muscle-tendon units, and muscle weakness [19]. Impaired motor control is observed in spastic CP, with lack of muscle coordination resulting from the initial neural insult. This is possibly due to impaired prediction of the sensory consequences of movement, leading to movement and overcorrection by co-activation of antagonist motor units [20].

The shortened muscle-tendon units of CP result from muscle cell growth dysfunction, leading to fewer sarcomeres, impaired regeneration, extracellular matrix expansion, and pro-inflammatory gene expression [21]. The shortened muscles ultimately produce weaker muscles as they are stretched across bones which grow at a normal linear velocity as a patient naturally develops.

**Figure 3:**
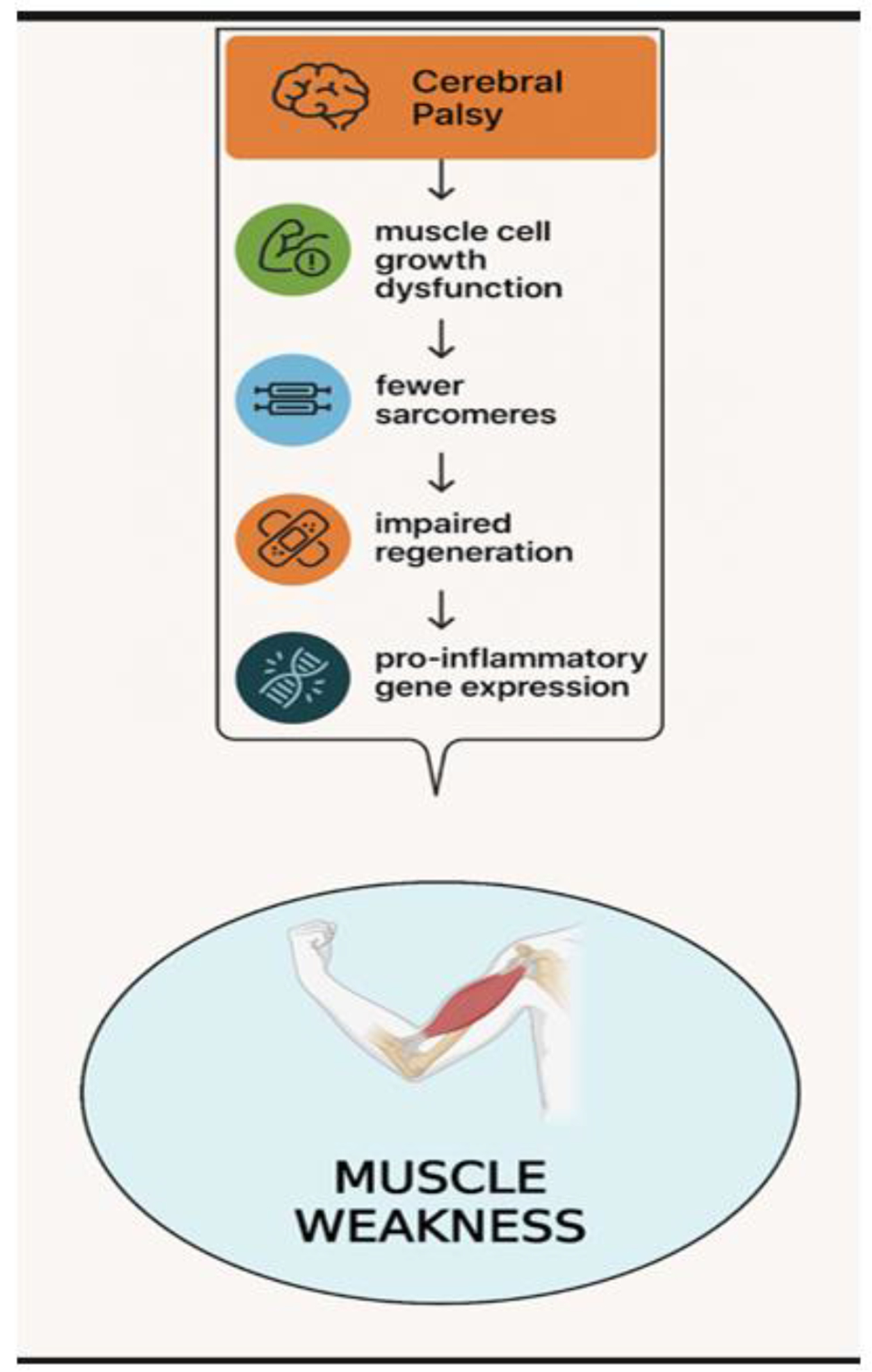
Cerebral Palsy patients experience muscle weakness following the shortening of their muscle tendon units. The pathophysiological changes that contribute to the muscle remodeling and growth dysfunction includes fewer sarcomeres, impaired regeneration of muscles, and pro-inflammatory gene expression.

Muscle weakness in CP can also be caused by deficient motor neuron recruitment, which is subsequently worsened by adaptive shortening from immobility and lack of mechanical loading from disuse [22]. Muscle weakness is also impacted by the reduced growth of sarcomeres and structural dysregulation of the muscle unit, as mentioned (Figure 3).

Muscle spasticity may in fact be a protective adaptive response to weakness and impaired motor control [20], but physiologically is due to altered force encoding in muscle spindles, leading to altered feedback gains and thresholds, causing abnormal reflexive contraction during normal movement [23]. As described, the understanding of the musculoskeletal pathogenesis of spastic CP is both intricate and dynamic to the natural development and growth as a patient age.

## 4. Diagnosis of Cerebral Palsy

Cerebral palsy cannot be diagnosed by any laboratory test or diagnostic imaging; its clinical presentation, as varied as it can be, makes the diagnosis [24]. Consistent with all diagnoses of CP is motor dysfunction that is disabling to the point of interfering with daily activities such as walking, running, jumping, or climbing. Thus, diagnosis is typically based on observations and parent reports with careful monitoring of motor milestones, deep tendon reflexes, and muscle tone [25].

Clinicians may find it helpful to classify CP by function severity using the Gross Motor Function Classification System (GMFCS), which defines five levels of gross motor function. These levels have been validated as corresponding to clinical expectations of motor development, are like the World Health Organization International Classification of Impairments, Disabilities and Handicap code, and have high inter-rater and physician-parent reliability [26]. For these reasons, much of the literature relies on these GMFCS levels to describe severity and prognosis of CP.

### 4.1 Complex Musculoskeletal Pathologies in Cerebral Palsy

Cerebral palsy presents with a wide range of neuromuscular deficits that frequently results in complex musculoskeletal deformities. These abnormalities can progress with age and impact the patient’s mobility, posture, and overall quality of life. This section focuses on the various complex musculoskeletal pathologies that can develop in patients including bony deformities, joint changes associated with spasticity and contractures, degenerative conditions, and neurological and pain syndromes observed with spastic CP.

## 5. Bony Deformities

### SPINE:

Patients with CP are prone to developing bony deformities of the spine, hips, knees, and ankles. Scoliosis is among the most common deformities of the spine, which can significantly impact the patients function and quality of life. It progresses rapidly during growth spurts and continues to progress even after skeletal maturity [27]. Patients often present with complaints of back pain and poor posture. On a physical exam, typical findings include asymmetrical shoulder height, prominence of one scapula, lateral trunk shift, or rib humps discovered on Adam’s forward bend test. Plain radiographs of the spine with sitting or standing can help identify the curve magnitude, known as the Cobb angle, and pelvic alignment which, depending on the severity of misalignment, tends to coincide with symptom severity. In cases where surgical intervention is being considered, advanced imaging such as computerized tomography and MRI can be used for additional information [28].

Other spinal deformities associated with CP include increased thoracic kyphosis, lumbar lordosis, and pelvic obliquity [27,28]. Pelvic obliquity in patients with CP is due to hip abductor weakness, scoliosis, hip displacement, asymmetric hip abduction, and limb length discrepancy which all combine to an imbalance of forces on the pelvis. This imbalance pushes it onto an oblique position leading to difficulty with gait [29–33].

### HIP:

Muscle tone imbalances can also lead to the subsequent hip deformities, which include femoral anteversion, apparent coxa valga, and windswept deformity [27,34]. Femoral anteversion often presents in CP during early adolescence and is due to an increase in the angle between the femoral neck at the proximal part of the hip relative to the femoral condyle at the distal point near the knee. This condition is believed to be caused by muscle forces pushing abnormally against the bone and altering the loading patterns during the femur in the growing stages [35]. Shefelbine showed that the mechanical loads in children with CP lead to altered stress and deformation patterns at the growth plate of the femur and results in the ensuing increased anteversion [36]. Bobroff also showed that healthy children exhibit a decrease in femoral anteversion with aging, while patients with CP do not, leading to their increased anteversion angles in adult life [37]. Patients can thus present with intoeing gait, excessive hip internal rotation, and limited external rotation leading to gait issues and potential falls [38].

Cerebral palsy patients also present with coxa valga which is characterized by the increase in neck to shaft angle of the femur which forms because of increased muscle forces and spasticity and affects the proper growth of the proximal femur in children [39]. The increased angle leads to the coxa valga phenotypic deformity and is associated with altered biomechanics and muscle imbalances. These include hip instability and subluxation, gait abnormalities, hip limited range of motion, and significant functional limitations. Patients categorized as GMFCS level IV and V who rely on assist devices for mobility often have one or more of these hip problems [27,39,40,41).

The presentation of windswept posture is attributed to the spasticity that affects the hip adductors and abductors asymmetrically, leading to the one hip that is adducted and internally rotated (Valgus deformity) with the other hip that is abducted and externally rotated (Varus deformity). Asymmetry that is prolonged secondary to immobility and the habitual lying position of patients with CP will lead to the worsening of the windswept hips as demonstrated by Ágústsson et al. [42]. There were further studies performed by Fulford et al. [43] that suggest that gravity also is a major factor on immobile individuals during early development leading to the development of this asymmetry [43]. Scoliosis and pelvic obliquity are prolonged postural asymmetries that further contribute to the strain patterns on the hips in CP patients which increase the likelihood of windswept deformities in children with CP [44]. Windswept deformity is more common in children with CP who have motor impairment and often present in the GMFCS level IV and V classification requiring assistance devices to have mobility.

### KNEE:

**Figure 4:**
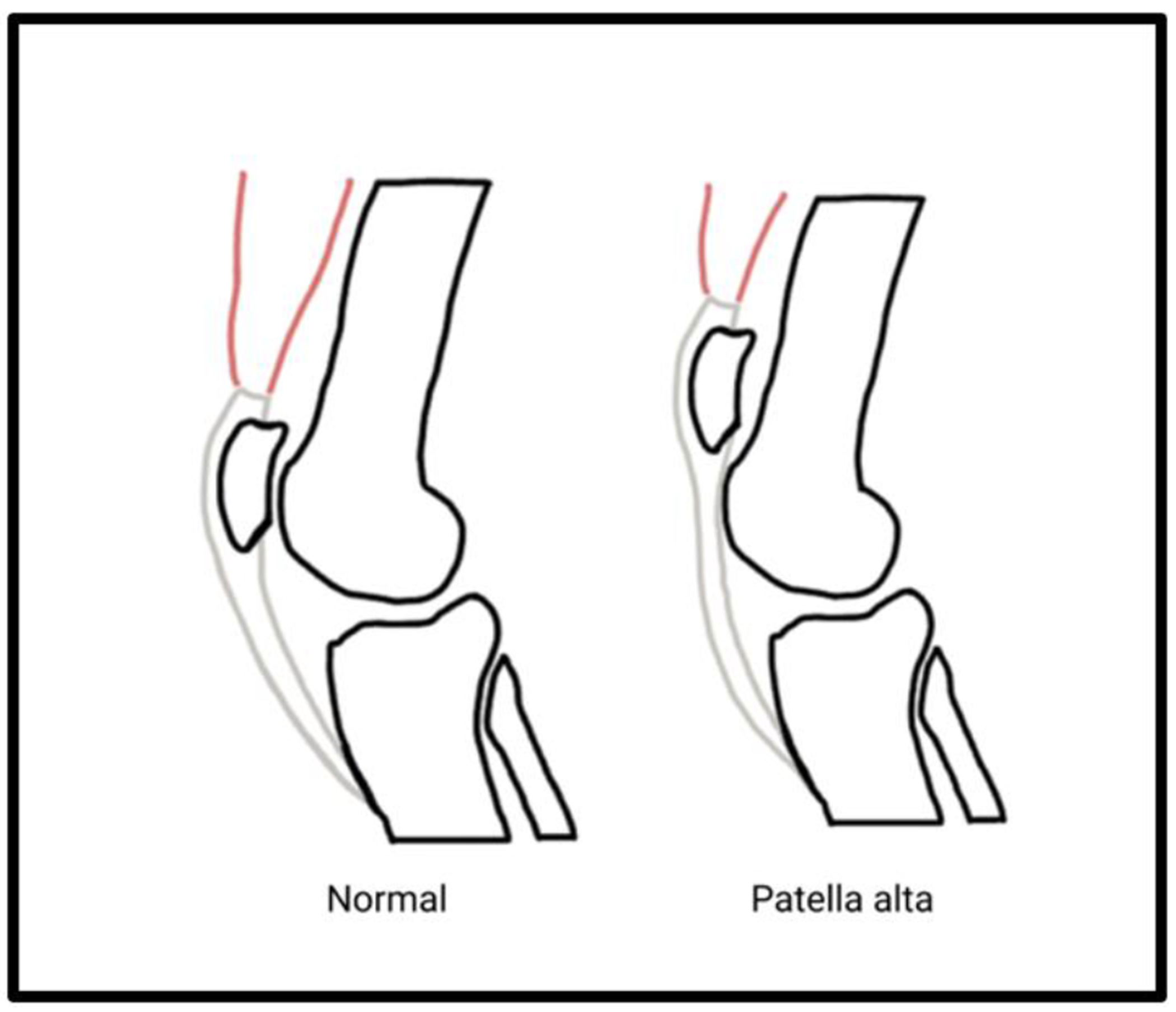
In patella alta, increased quadriceps activation contributes to chronically increased superior force on the patella, repositioning the bone. This higher patellar position causes knee instability and pain.

Patella alta and patellar fragmentation are conditions observed in patients with CP and lead to subsequent knee pain and instability [27]. Patella alta is characterized by an abnormally high positioning of the patella that is common in CP, especially patients who walk with excessive ankle dorsiflexion, knee flexion, and hip flexion during the stance phase also known as crouch gait. The increased quadriceps activation as well as patellar tendon forces during crouch gait can produce an abnormally high riding patellar position [45,46]. These patients can clinically present with anterior knee pain and knee instability with increasing lateral displacement and tilt on the patella as well as a predisposition to patellofemoral misalignment [47,48] (Figure 4).

Patellar fragmentation at the distal pole of the patella is thought to be primarily due to the excessive tension in the quadriceps mechanism, often with a coexisting flexion contracture of the knee that leads to an abnormal stress and loading on the patella and subsequent fragmentation [49]. It can be clinically mistaken for Osgood-Schlatter disease due to the location of the knee pain, but the radiographic evidence of the inferior patellar pole fragmentation along with the bone marrow edema-like signals on MRI which makes it not a normal variant and a distinct pathology [50].

Additionally, the recurvatum deformity, which results from repeated contracture of the rectus femoris muscle, can lead to hyperextension of the knee during the stance phase of gait which exacerbates the instability and mechanical stress on the joint. The deformity occurs when the tibia halts anterior motion relative to the femur and creates an extension momentary position in the knee. Excessively active calf muscles when the ankle is in dorsiflexion will also lead to the arrest of forward movement of the tibia and worsen the recurvatum deformity [51]. Additionally, patients with equinus foot deformity, which is common in CP, will further exacerbate the recurvatum deformity as limitation to the ankle dorsiflexion will lead to an even greater need for knee hyperextension to compensate when ambulating [52].

### ANKLE:

Over 90% of patients with CP will develop a foot or ankle deformity at some point in the disease course [53,54]. Some common variations include equinovarus, planovalgus, cavovarus, equinus, equinocavovarus, and equinoplanovalgus, with the latter three being the most common [55,56]. The classification of these deformities is largely descriptive, but the pathology in each case follows from some combination of pathologically spastic muscle groups [57]. The literature suggests that valgus deformities are associated with higher GMFCS scores, which may be a useful prognostic factor for clinicians [58].

Equinus presents as marked plantarflexion of the ankle, usually due to spasticity in gastrocnemius and soleus. Equinovarus combines this plantarflexion with inversion of the foot due to spasming of the tibialis posterior. Cavovarus combines foot inversion with arch exaggeration, while planovalgus combines foot eversion with arch flattening. Equinoplanovalgus combines plantarflexion with eversion and arch flattening, while equinocavovarus combines plantarflexion with inversion and arch exaggeration. Radiographic criteria are available for many of these conditions, but they may be grossly evident on examination.

Two additional deformities should be noted because of their clinical significance. Ankle valgus, which is defined by a tibiotalar angle of 10° or more, is associated with cavus deformity and equinovalgus foot [54]. Hallux valgus is the deformity of the first metatarsophalangeal joint resulting in medial deviation of the metatarsal, which develops due to a combination of muscle spasticity and altered mechanical patterns [54,59]. When present, these deformities may contribute significantly to pain and disability.

## 6. Joint Changes (Contractures and Spasticity)

The changes at the joint level in spastic CP result from the muscle spasticity and contractures, with the spine and lower extremities being the most commonly affected. Contractures at the joint result from the muscle spasticity, which produces a permanent shortening of the muscle-tendon units and leads to the various joint deformities. The development of contractures is proposed to occur secondarily to the increased stiffness of the muscles and extracellular matrix. Patients with CP demonstrate increased stiffness of the muscles that is associated with abnormally stretched sarcomeres and increased stiffness of the extracellular matrix due to the increased collagen content which contributes to muscle rigidity and reduced muscle elasticity [60,61]. The recognition and early interventions for contractures are critical to stop the progression and eventual irreversible changes to the joint space which can make it difficult to perform activities of daily living.

### SPINE:

Spinal deformities at the joint level, including scoliosis, are worsened depending on the severity of motor impairment. Patients with high GMFCS levels (IV or V) show significantly higher annual increases in scoliosis Cobb angle, thoracic kyphosis angle, and apical vertebral translation [62]. In addition, postural asymmetries have a strong association with scoliosis and other spinal joint deformities which are worsened by severe motor impairments [63]. Factors that contribute to the likelihood of scoliosis and contractures include age, postural asymmetry, and high GMFCS levels [44]. The reduction of spinal inhibition of motoneurons from sensory pathways in patients with CP further contributes to the spasticity and impaired motor function [64].

### HIP:

**Figure 5:**
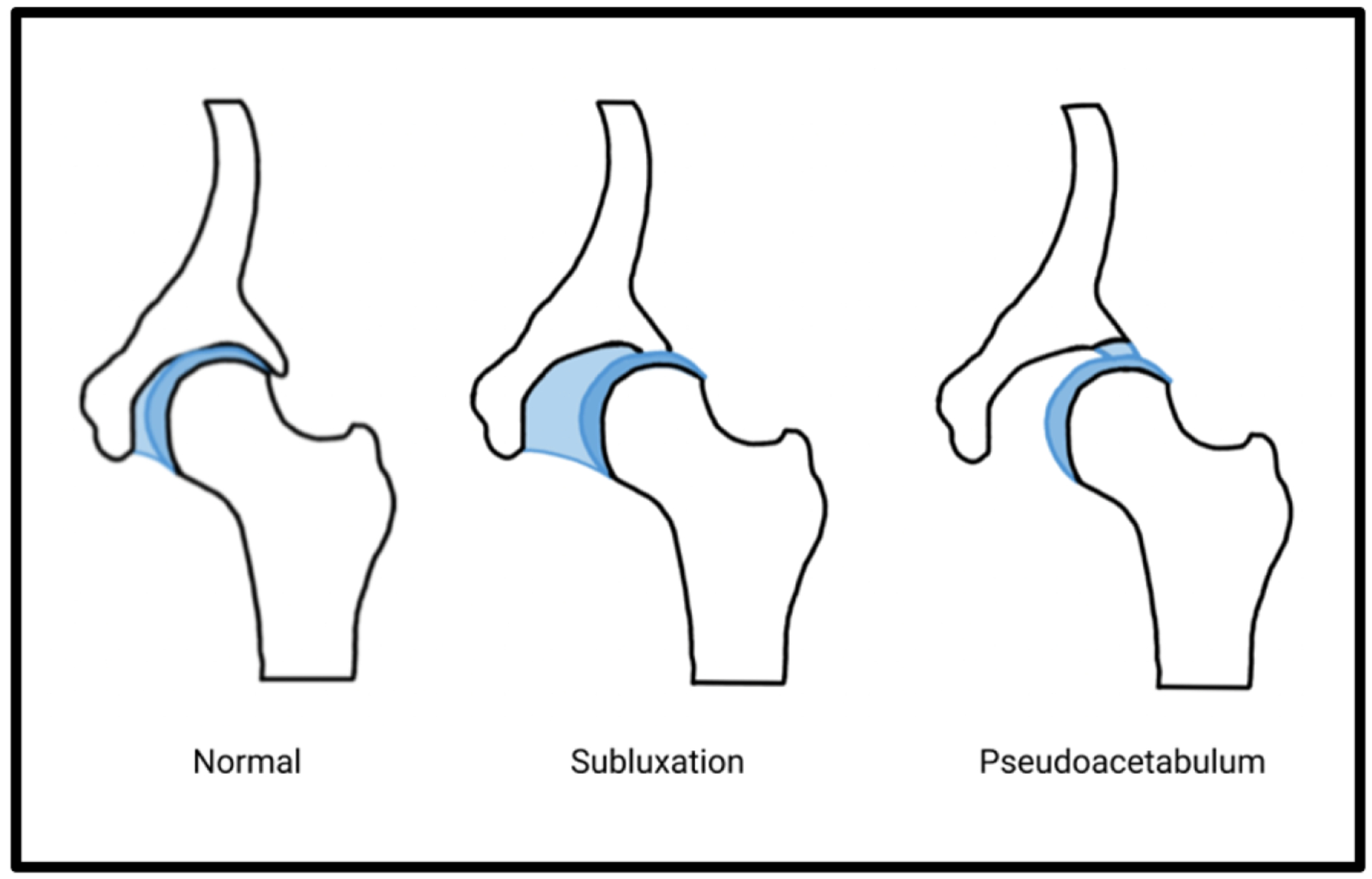
In pseudoacetabulum, progressive hip subluxation, dislocation, and joint remodeling produces a new erosive articulation with the ilium.

The hip is prone to subluxation and dislocation, especially in non-ambulatory patients secondarily to the spasticity and muscle imbalances in patients with CP. The imbalance of forces between counteracting muscles will lead to progressive hip displacement, with risk for both subluxation and displacement especially in non-ambulatory patients or those with severe spasticity [65,66]. The progressive joint remodeling can also lead to pseudoacetabulum formation which is secondary to chronic hip subluxation and dislocation in CP (Figure 5). The femoral head will become repositioned outside the true acetabulum, erode the articular cartilage, and create a new articulation with the ileum which produces the pseudoacetabulum [67]. The hip joint in these patients can no longer be classified as a true joint but a secondary articulation site due to the joint morphogenesis that is plagued with significant joint incongruity and instability [68,69]. Patients will typically complain of localized pain due to the degeneration of the joint and have difficulty with sitting and perineal care due to the altered hip mechanics [70,71].

### KNEE:

The knee is prone to flexion contractures leading to limitation in extension and significant functional impairments [72,73]. The spasticity and reduced range of motion is linked to the spasticity of the hamstrings and gastrocnemius muscles which lead to reduced knee extension and ankle dorsiflexion, further impairing the walking ability and knee functions [74].

## 7. Spinal Degeneration and Damage

The various spastic forces on the spine of CP patients often can lead to abnormal postural positions and biomechanical movements. Over time, the compounded forces can predispose the patients to permanent degenerative changes of the spine, including spinal stenosis, disc herniations, and postural instability [75].

### CERVICAL SPINE:

The hyperexcitability of the brain stem pathways in CP patients can increase muscle tone and spasticity around the cervical spine which further places stress on the spinal column leading to conditions such as cervical stenosis [75,76]. Additionally, patients with a congenital or developmental predisposition for cervical stenosis can have the process of spinal canal degeneration and narrowing be accelerated by the increased mechanical forces placed on the spine in spastic CP patients, leading to the symptomatic cervical spinal stenosis presentation at an earlier age [77]. Cervical stenosis is observed in 7.5% of adults with spastic CP from studies done between 2006 and 2016 [75]. The patients with cervical stenosis often present with upper extremity symptoms (73%), neck pain (53%), ambulation decline (70%), and incontinence (30%) [75].

Disc herniations in the cervical spine can also be caused by the sustained abnormal tonicity and abnormal movements of the neck seen in CP patients [78]. The literature also suggests that CP predisposes patients to cervical spondylosis with disc protrusions, further contributing to the cervical disc herniations [79]. The disc herniations can lead to myelopathy with a rapid deterioration of neurological function including weakness, numbness, and loss of coordination.

### LUMBOSACRAL SPINE:

At the lumbosacral spine, most often at the L5-S1 level, spondylosis and spondylolisthesis is a frequently encountered complication of CP. This is commonly seen in ambulatory adult patients who have spastic diplegia [34]. In fact, if lumbar spondylolysis is not diagnosed early, the condition can progress to spondylolisthesis, leading to further spinal instability and pain. The mechanical forces on the spine, including the vertical loads, muscle activity, and effects of movement, contribute to the development of spinal degenerative conditions [80]. Spondylolysis and spondylolisthesis can occur when these forces create an anterior shear stress which is placed upon and resisted by the annulus fibrosus, facet joints, and pars interarticularis [80]. Further investigation by Sakai et al demonstrated that the lumbar spinal spondylolysis and stress related spinal processes were more common in patients with mixed CP, described as a spastic CP with athetoid movements (dyskinetic movements) [81]. Specifically, if there was combined motion of extension and axial rotation on the spine, as seen in the biomechanical analysis on patients with CP, it produced a higher stress at the pars interarticularis, particularly at the L5 level, leading to the shearing that contributes to the spondylolysis and spondylolisthesis.

## 8. Neuromuscular Dysfunction

CP patients can struggle with episodes of pain from a multitude of conditions. At the spinal level, scoliosis and abnormal hypercurvatures can place extra stress and load at the joint level which leads to a feeling of discomfort [82]. Children with CP often have asymmetries of posture with sitting and supine positions that produce these abnormal spinal positions and lead to subsequent pain [83]. Reduction in mobility and limited ability to perform position changes can further worsen the pain [84].

Hip pain is common and often associated with hip displacement, and the severity of the pain is often related to the degree of hip displacement [85,86]. The intensity of the pain is also associated with the presence of degenerative cartilaginous lesions [87]. CP patients have an increased susceptibility to develop hip and knee arthritis secondary to the local stress concentrations that damage the articular cartilage and lead to early joint degeneration and osteoarthritis [68]. The reduction in contact pressure at the joints can also precipitate a thinner cartilage surrounding the bone as well as osteopenia which further accelerates the degeneration and pain at hips and knees [68].

Weakness at the hip and knee joint levels occurs primarily due to the neuromuscular impairments that arise from early brain injury. The ensuing impairments include short muscle-tendon units and impaired selective motor control [19]. Studies have shown that in comparison to children without CP, those with CP have significantly weaker limb muscles, with the knee extensors being the most affected muscles [88,89]. These individuals have a much harder time keeping an upright posture and stability with gait. They also produce a reduced stride length, slower walking speeds, and form a higher reliance on muscular contributions to maintain the leg stiffness needed to perform stable gait [90, 91].

## 9. Conclusions

Cerebral palsy is a common and highly varied condition that causes a wide range of complications and subsequent disability worldwide. The diagnosis of CP relies on the clinician’s ability to understand and recognize its varied presentation as a patient age and develops. The aim of our review is to consolidate the diverse presentations and etiologies of common lower extremity pathologies seen in spastic CP. Defects of the lower extremity and spine hinder a patient’s ability to crawl, stand, walk, run, jump, and climb at every developmental milestone, which produces difficulty in completing activities of daily living for patients with CP. Furthermore, our focus on the musculoskeletal aspects highlights variations that can be grossly appreciated during the physical examination. Lastly, CP is a multisystem disorder, and pain is another disabling factor of its presentation. Recognizing the full scope of musculoskeletal complications in spastic CP will give providers a framework through which they can understand and appreciate the multifarious presentation of the patient with CP.

## 10. Future Considerations

Our review consolidated numerous studies and documentations of musculoskeletal complications of CP to lay the groundwork for further discussion of management. As CP is highly variable, its medical and surgical management must also be diverse and dynamic. As more patients with CP are living longer and entering adulthood, providers must be able to diagnose the various complications to guide appropriate treatment with the goal of improving independence and self-sufficiency. With our integrated review of the major musculoskeletal complications of the lower extremity in patients with spastic CP, we hope that this framework can be leveraged to similarly explore and consolidate the multimodal approaches to treatment and management of CP.

## 11. Key Points

- **Definition and prevalence**: Cerebral palsy (CP) is the most common childhood disability, affecting 2-3 per 1000 live births in the US, characterized by permanent, non-progressive motor and postural disturbances due to early brain development issues.
- **Risk factors:** CP risk factors include antenatal (e.g., brain malformations), perinatal (e.g., hypoxic-ischemic encephalopathy), and postnatal (e.g., infections) causes, with maternal factors like advanced age, high BMI, pregestational diabetes, and multiple gestations increasing predisposition.
- **Presentation:** Spastic CP, the most common subtype, is associated with four key neuromuscular impairments: impaired motor control, muscle spasticity, shortened muscle-tendon units, and muscle weakness.
- **Pathophysiology of MSK complications**: Musculoskeletal complications include impaired motor control from neural insults, shortened muscle-tendon units due to growth dysfunction, and muscle weakness from poor motor neuron recruitment and disuse, with spasticity possibly serving as a protective response.
- **Spinal deformities:** Scoliosis is prevalent in CP, progressing with growth spurts, alongside thoracic kyphosis, lumbar lordosis, and pelvic obliquity, driven by muscle imbalances and spasticity, affecting posture and mobility.
- **Hip deformities:** Femoral anteversion, coxa valga, and windswept deformity arise from spastic muscle forces, leading to gait abnormalities, instability, and functional limitations, especially in GMFCS IV-V patients.
- **Knee and ankle deformities:** Knee conditions (e.g., patella alta, recurvatum) and ankle deformities (e.g., equinus, equinovarus) result from spasticity and contractures, causing pain, instability, and gait issues, with over 90% of CP patients developing foot/ankle deformities.
- **Joint deformities:** Spasticity and contractures cause joint deformities (e.g., hip subluxation, knee flexion contractures) and spinal degeneration (e.g., cervical stenosis, spondylolisthesis), worsened by mechanical stress and motor impairment severity.
- **Neuromuscular dysfunction:** Pain in CP stems from spinal deformities, hip displacement, and early arthritis, while weakness, particularly in knee extensors, arises from neuromuscular impairments, reducing mobility and stability in gait.
